# Functional thalamocortical connectivity at term equivalent age and outcome at 2 years in infants born preterm

**DOI:** 10.1016/j.cortex.2020.09.022

**Published:** 2021-02

**Authors:** Hilary Toulmin, Jonathan O'Muircheartaigh, Serena J. Counsell, Shona Falconer, Andrew Chew, Christian F. Beckmann, A. David Edwards

**Affiliations:** a Centre for the Developing Brain, Division of Imaging Sciences and Biomedical Engineering, King's College London, St Thomas' Hospital, London SE1 7EH, UK; b Neurodevelopmental Service, Brookside Family Clinic, Cambridge and Peterborough NHS Foundation NHS Trust, 18 Trumpington Road, CB2 8AH, UK; c Cambridgeshire Community Services NHS Trust, Peacock Centre, Brookfields Hospital, Cambridge, CB1 3DF, UK; d Department of Forensic and Neurodevelopmental Sciences, Sackler Institute for Translational Neurodevelopment, Institute of Psychiatry, Psychology and Neuroscience, King’s College London, UK; e MRC Centre for Neurodevelopmental Disorders, King’s College London, London, UK; f Donders Institute for Brain, Cognition and Behaviour, Radboud University, 6500 HC, Nijmegen, the Netherlands; g Department of Clinical Neuroscience, Radboud University Medical Centre, 6500 HB, Nijmegen, the Netherlands; h Oxford Centre for Functional Magnetic Resonance Imaging of the Brain (FMRIB), University of Oxford, Oxford, OX3 9DU, UK; i Department of Bioengineering, Imperial College London, London, SW7 2AZ, UK

**Keywords:** Functional MRI, Preterm infants, Thalamocortical connectivity, Cognitive outcome, Motor outcome

## Abstract

Infants born preterm are at high risk of long-term motor and neurocognitive deficits. In the majority of these infants structural MRI at the time of normal birth does not predict motor or cognitive outcomes accurately, and many infants without apparent brain lesions later develop motor and cognitive deficits. Thalamocortical connections are known to be necessary for normal brain function; they develop during late fetal life and are vulnerable to perinatal adversity. This study addressed the hypothesis that abnormalities in the functional connectivity between cortex and thalamus underlie neurocognitive impairments seen after preterm birth. Using resting state functional connectivity magnetic resonance imaging (fMRI) in a group of 102 very preterm infants without major focal brain lesions, we used partial correlations between thalamus and functionally-derived cortical areas to determine significant connectivity between cortical areas and thalamus, and correlated the parameter estimates of these connections with standardised neurocognitive assessments in each infant at 20 months of age. Pre-motor association cortex connectivity to thalamus correlates with motor function, while connectivity between primary sensory-motor cortex and thalamus correlates with cognitive scores. These results demonstrate the importance and vulnerability of functional thalamocortical connectivity development in the perinatal period for later neurocognitive functioning.

## 1 Introduction

Preterm birth is the leading cause of child deaths in high-income countries (Litt et al., 2012) and has lifelong neurodevelopmental effects and increased risk of chronic disease stretching into adulthood (Aarnoudse-Moens, Weisglas-Kuperus, van Goudoever, & Oosterlaan, 2009; Boardman et al., 2010; Dyet et al., 2006; Linsell, Malouf, Morris, Kurinczuk, & Marlow, 2015; Nagy, Lagercrantz, & Hutton, 2011; Nosarti et al., 2014; Wood et al., 2005). Infants born extremely preterm (22–27 weeks gestational age) represent the severe end of a spectrum of health and developmental adversity with 57–72% of survivors experiencing motor, cognitive or language impairments by secondary school or adolescence (Hutchinson et al., 2013; Johnson & Marlow, 2017; Marlow, 2004; Moore et al., 2012; Saigal & Doyle, 2008).

T1 and T2 weighted MR imaging at the time of normal birth is not precising in detecting infants who develop later neuromotor or neurocognitive problems, failing to detect around 1/3 of the children with impairments (Edwards et al., 2018). These infants who do not have major structural abnormalities at birth are also at high risk of motor (Grunewaldt et al., 2014; Spittle et al., 2013), cognitive and language (Larroque et al., 2008; Moore et al., 2012), social, emotional and psychiatric problems (Delobel-Ayoub et al., 2006; Kuzniewicz et al., 2014), difficulties which persists into late childhood and adult life (Akshoomoff et al., 2017; Johnson et al., 2009; Linsell et al., 2018; Litt et al., 2012; Northam et al., 2012; Nosarti et al., 2012). The complexity of these impairments, without obvious macrostructural predictors, argues for fundamental changes in brain circuitry and connectivity, which may be reflected in measures of resting state functional connectivity.

Functional connectivity between thalamus and cortex develops rapidly between 30- and 40-weeks’ gestational age and has been shown to be disrupted in preterm infants using functional magnetic resonance imaging (fMRI) (Toulmin et al., 2015). We hypothesised that abnormal functional thalamocortical connectivity leads to long-term neurocognitive impairment after preterm birth. We performed fMRI assessments of functional thalamocortical connectivity in 102 preterm infants born at less than 33 weeks’ gestation, together with data on neurocognitive abilities at 20 months of age in order to address the question: does the strength of thalamocortical connectivity at the time of normal birth correlate with motor, cognitive or language capabilities at the age of 20 months in a cohort of infants without structural abnormalities?

## 2 Materials and methods

The work was reviewed and approved by the National Research Ethics Service (UK) and all infants were studied with written consent of their parents obtained according to the Declaration of Helsinki. Over a period of three years 2010–2013, preterm participants were recruited at birth as part of the Evaluation of Preterm Imaging Study (e-Prime) (NCT01049594) from hospitals in the North and Southwest London Perinatal Network (Edwards et al., 2018). Eligibility included birth before 33 weeks gestational age to a mother who was aged over 16 and able to speak English. Infants were excluded if they had major congenital abnormalities or metallic in-plants. Full eligibility criteria can be found in Edwards et al., 2018. MRI were carried out at a single neonatal imaging centre when the infant was a gestational age of 38–44 weeks. The neurodevelopmental outcomes of these 511 infants are typical of populations in major studies of preterm outcomes, such as EPIPAGE (Larroque et al., 2008).

### 2.1 Imaging acquisition

All images were acquired on a 3 T Philips Achieva MRI scanner (Best, Netherlands) under sedation using oral chloral hydrate 25–50 mg kg (Finnemore et al., 2014), supervised by an experienced paediatrician. Pulse oximetry, temperature and electrocardiography data monitored throughout. Ear protection was provided with silicone-based putty placed in the external ear (President Putty, Coltene, Whaledent, Mahwah, NJ) and Minimuffs (Natus Medical Inc, San Carlos, CA).

Whole-brain functional imaging was performed using a T2∗ gradient echo planar image acquisition (sequence parameters: TR = 1.5 sec; TE = 45 ms; flip angle = 90°, 256 volumes, slice thickness 3.25 mm, in-plane resolution 2.5 mm^2^, 22 slices, scan duration = 6.4 min) with an 8 channel phased array head coil. T2-weighted fast-spin echo MRI was acquired using TR: 8670 ms; TE: 160 ms; flip angle 90°; slice thickness 2 mm; field of view: 220 mm matrix: 256 × 256 (voxel size: .86 × .86 × 1 mm).

### 2.2 Cohort

We report how we determined our sample size, all data exclusions, all inclusion/exclusion criteria, whether inclusion/exclusion criteria were established prior to data analysis, all manipulations, and all measures in the study as follows. All inclusion/exclusion criteria were established prior to data analysis. The 511 infants recruited into the EPRIME cohort were born at less than 33 weeks completed gestation (GA) and were scanned once at term equivalent age. 275 infants had full MRI including resting state functional connectivity data and had complete follow-up (including MCHAT). Datasets with a relative mean displacement of >.08 mm were excluded as were remaining datasets with absolute motion of >2 mm. 15 further subjects were excluded as they had been scanned at > 44 weeks postmenstrual age. Of the remaining 107 infants, 5 had structural brain abnormalities, defined as abnormality scores of greater than 12 (categorised as severe) according to criteria in Woodward et al., 2006. There were therefore 102 preterm infants suitable for functional connectivity analysis. Table 1 gives clinical and demographic data for this cohort of 102 infants.

**Table 1:** Demographic details of the cohort.

Mean gestational age in weeks at birth (range)	30 (24.43–32.86)
Mean birth weight in grams (range)	1360.3 (600–2510)
Mean numbers of days ventilated (range)	1.74 (0–16)
Mean gestational age (in weeks) at scan (range)	41.9 (38.29–44)
Mean score for prognosis on term MRI scan (range) (Woodward et al., 2006). no abnormality = 9 mild abnormality = 79 moderate abnormality = 14.	8 (5–12)
Mean gestational age in months at neurodevelopmental follow up	19.91 (19–23)
Sex (% female)	44 (45 female, 57 male)
Gross Motor composite score (range)	96.10 (range 61–118)
Cognitive composite score (range)	94.22 (range 65–130)
Language score (range)	16.67 (4–31)

There was no relationship between gestational age at birth and motion (*r* = .03, df 100, *p* > .5) or between motion and the three variables of motor, cognitive or language scores: motor score and motion (r = .01, df 100, *p* > .5), cognitive score and motion (*r* = .088, df 100, *p* > .5) or language score (*r* = .134, df 101, *p* = .179).

### 2.3 Image processing

The first six volumes of each subject were removed to allow time for equilibration of T1 magnetisation, and motion correction using MCFLIRT was applied (Jenkinson, Beckmann, Behrens, Woolrich, & Smith, 2012). Single subject ICA was performed on each dataset with automatic dimensionality using MELODIC (Beckmann & Smith, 2004) high pass temporal filtering of 125s, without slice-timing correction or spatial smoothing to preserve the high-frequency spatial and temporal signal. This was followed by FIX (FSL) for automatic de-noising and artefact removal (Salimi-Khorshidi et al., 2014). The standard FIX processing steps involved masking of datasets in standard space using the adult MNI152 atlas was modified to use a population-specific neonatal template with tissue priors rather than registration to the adult MNI152 atlas (Serag et al., 2012). MELODIC provides components which undergo automatic component classification by FIX, which allows the unique variance of each noise component to be regressed out of the data alongside the full variance of the motion parameters and derivatives (Salimi-Khorshidi et al., 2014; Satterthwaite et al., 2012). This has been shown to be an effective method in infants (Ball et al., 2016).

### 2.4 Group ICA

**Fig. 1:**
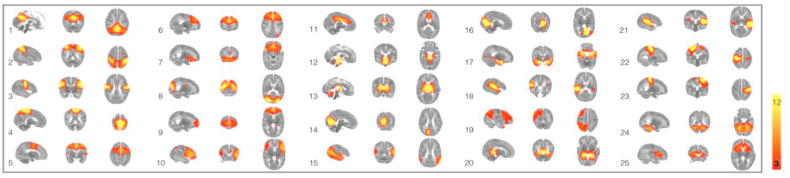
Temporal concatenation ICA-estimated resting pattern in the group of 102 subjects. Sagittal, coronal and axial views of the spatial map for each component. Images are z statistics overlaid on the template 41-week brain. Red to yellow indicates z values ranging from 3 to 12. The right hemisphere of the brain corresponds to the left sides of the coronal and axial images. Components 1–11 correspond to functional cortical components described in Table 3.

To generate functionally defined regions of interest in each subject to use for seed–based correlation analysis, the following process was followed. Functional volumes were registered to the subject's T2-weighted structural image (Jenkinson, Bannister, Brady, & Smith, 2002) with boundary-based registration (Greve & Fischl, 2009) as implemented by FMRIB's boundary-based registration (FSL-BBR) optimised for neonatal tissue contrasts. These images were then transformed to a population-based neonatal template (Kuklisova-Murgasova et al., 2011) using nonlinear registration (Jenkinson et al., 2012). It is not possible to reliably identify single voxels of cerebrospinal fluid from the echo planar image of an infant at the time of normal birth to model time course regressors as is done in adult studies, as ventricles are too small to avoid partial volume effects. Therefore, a mask of cerebrospinal fluid, defined using the high-resolution image, was applied to individual datasets at this point and data from voxels corresponding to these areas were discarded. Cortical regions of interest were defined using components from independent component analysis as follows. Pre-processed functional data containing 250 contiguous time points per subject were temporally concatenated across subjects to produce a single 4D data set and resting state components common to the group were defined using MELODIC (Beckmann, DeLuca, Devlin, & Smith, 2005) with a fixed dimensionality of 25 which achieves a good balance between interpretability and robustness as used in adults datasets (Smith et al., 2012). ICA maps were thresholded using an alternative hypothesis test based on fitting a Gaussian/Gamma mixture model to the distribution of voxel intensities within spatial maps and controlling the local false discovery rate at *p* < .05 (Beckmann et al., 2005). The resulting maps and full ICA decomposition are shown in Fig. 1.

### 2.5 Cortical component selection

**Fig. 2:**
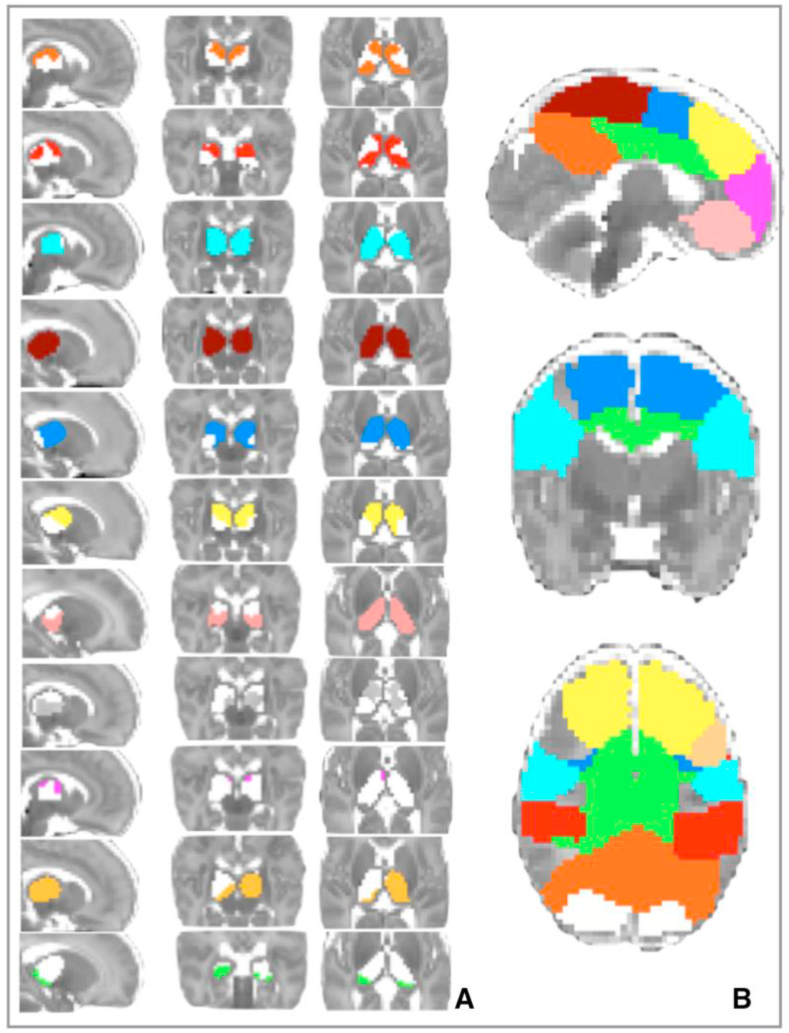
Significant correlations between thalamus (A) and 11 cortical regions of interest (B) in 102 preterm infants at the time of birth. Partial correlations (group mean) are thresholded at a significance of *p* < .05 (family-wise error corrected). Colours and order as per Table 3. Images are displayed as per radiological convention.

Non-overlapping cortical areas were defined by assigning each voxel to a specific resting state component depending on which network had the highest z-score at that voxel. The result is shown in Fig. 2. The spatial correlation with adult networks was tested using cross correlations after registration of infant data to an adult template and with previous work in preterm and term infants and the results shown in Table 2. As there is no clear consensus as to how to interpret laterality at the time of birth, and the underlying architecture of thalamocortical connectivity is of reciprocal connections with inter hemispheric cortico-cortical reciprocity (Jones, 2007; Sherman & Guillery, 2013), it was decided to model bilateral cortical inputs to the thalamus. In addition, this allows the use of a cortical parcellation based on ICA and unbiased by assumptions of future adult function. Consequently, the 11 cortical areas with bilateral hemispheric representations in the group ICA were used for further analysis. Of note, therefore, are the omission of the unilateral components, all of which had symmetrical counterparts in the contralateral hemisphere: numbered 14 and 16 (medial visual), 18 and 21 (auditory) and 22 and 23 (sensory-motor lateral portion). The mask for the thalamus was created using a neonatal-specific template (Makropoulos et al., 2014) and includes right and left thalami. Voxels in cortical components adjacent to subcortical seed masks were removed to ensure that no cortical component was directly adjacent to the thalamus mask.

**Table 2:** Cortical components used in the analysis and their anatomical correlations with adult (Smith et al., 2012) and infant (Toulmin et al., 2015) resting state networks.

	Infant resting state network in cohort two (numbers as per Tab 8.1)	Corresponding adult resting state network (Smith et al., 2012)	Correlation with Smith et al., 2012	Correlations with infant resting state networks in cohort of 66 term and preterm infants (Toulmin et al., 2015)
1	Posterior cingulate	16	.44 (also 17 .40)	.6, .52
2	Lateral parietal	12	.35	.8
3	Fronto-parietal-insula	7	.65	.62
4	Primary sensory motor (medial)	5	.56	.46
5	Motor association	9	.39	.69
6	Superior frontal	15	.43	.68
7	Orbito-frontal	18	.32	.69
8	Visual	2	.63	.34
9	Pre-frontal	18	.54	.61
10	Lateral pre-frontal	20	.49	.38
11	Anterior cingulate	16	.28	.5

Within a group-defined cortical functional area, there is likely to be some heterogeneity at the subject level. For each individual subject, each component identified at the group level was mapped back to each subject's data set through a spatial regression of the group ICA maps on the individual fMRI dataset, followed by a regression of the resulting time series on the same dataset (Filippini et al., 2009). To ensure that the first eigentimeseries at the subject specific level best represented the function determined by the group analysis rather than to another functional area within the same group defined cortical region, for each subject, the components were thresholded at *z* = 1.96 and the remaining voxels used as the cortical target from which the first eigentimeseries was defined. The resulting component maps in individual subjects derived using this dual regression approach were used as regions of interest and for each thalamic voxel, partial correlation between this voxel and all the cortical eigentimeseries were calculated (O'Reilly, Beckmann, Tomassini, Ramnani, & Johansen-Berg, 2010). The cortical regions analysed are described in Table 3.

**Table 3:**
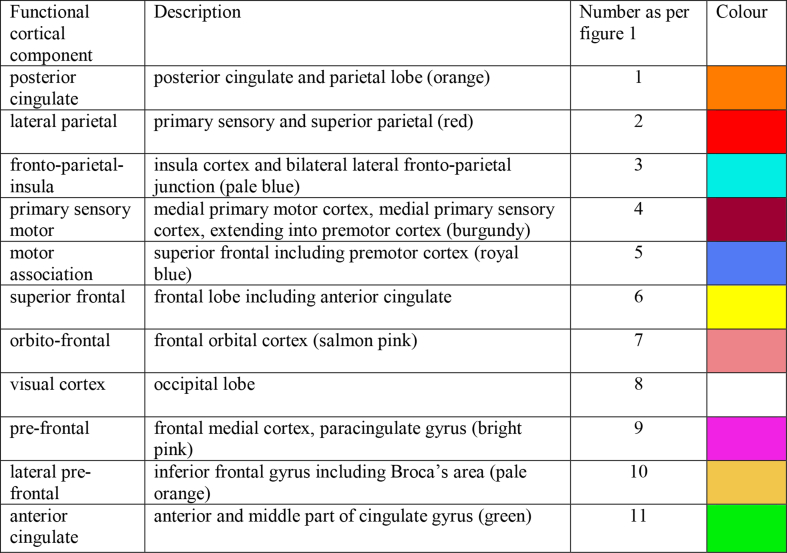
11 bilateral regions of interest: functional cortical components defined using independent component analysis in the cohort of 102 preterm infants (full list of 25 components from the ICA in Fig. 1).

### 2.6 Neurodevelopmental examination

Neurological and developmental testing was performed at 20 months of age, corrected for gestational age at birth, using the Bayley Scales of Infant and Toddler Development, Third Edition known as Bayley-III (Bayley, 2005) by researchers experienced in neurodevelopmental testing. The Bayley III scores allow comparison of children tested at different ages and have a mean of 100 with a standard deviation of 15. The average scores for this cohort are motor composite score 96.10 (range 61.00–118.00), cognitive composite score 94.22 (range 65.00–130.00) and language sum scaled score 16.67 (range 4.00–31.00).

### 2.7 Group analysis

**Fig. 3:**
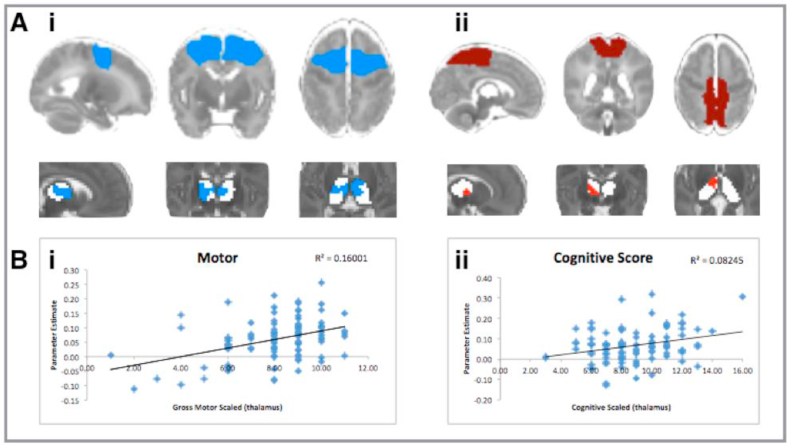
Significant correlations with outcome at 2 years. (Ai) shows the bilateral locations in the thalamus where the correlation coefficients with the premotor region of interest at the time of normal birth are significantly correlated with motor outcome at 2 years old. (Aii) shows the area of the right thalamus where the correlation coefficients with the sensory-motor region of interest at the time of normal birth is correlated with cognitive scores at 2 years old. (B) Scatterplots showing the association between correlation scores between thalamus and cortical area (vertical axis) and outcome score for that subject (horizontal axis). Images are shown as per radiological convention. Significance testing using randomisation is shown at *p* < .05 (family-wise error corrected) using a general linear model to determine the relationship between correlation coefficients at the time of normal birth and outcome at 2 years old. In addition to family-wise error correction, these results also retain significance with voxel-wise testing. Cortical regions of interest and thalamus statistical images are shown in the same colours as per Table 3.

Using a general linear model which includes gestational age at birth, gender and age at scan as explanatory variables, the corticothalamic correlations were tested voxel-wise for statistically significant associations with neurodevelopmental scores at 2 years of age using nonparametric permutation testing (Nichols & Holmes, 2002) tested at a significance *p* < .05 corrected for multiple comparisons after threshold-free cluster enhancement (Smith & Nichols, 2009). This resulted in spatial maps characterising the effect of prematurity on connections between cortical areas and thalamus (Fig. 3).

## 3 Results

Fig. 1 shows the temporal concatenated ICA-estimated resting pattern in the group of 102 preterm infants, from which the cortical regions of infants were defined. Hard-thresholding the functional connectivity estimates of the 11 functionally-defined cortical areas (Table 3) revealed a predominantly symmetrical topographical representation of these cortical regions in the thalamus at the time of normal birth (Fig. 2), in line with previous work (Cai, Wu, Su, Shi, & Gao, 2017; Ferradal et al., 2019; Toulmin et al., 2015). The exception to this is the lateral prefrontal component which has a larger cortical representation on the left in the group independent component analysis (Fig. 1) and a predominantly left-sided representation in Fig. 2. The cortical areas including primary motor cortex and supplementary cortex (cortical component 4 -royal blue and cortical component 5 burgundy), both have widespread connectivity throughout the thalamus, with the contribution of cortical component 5 being slightly more restricted.

The scoring of structural MRI at birth using criteria described by Woodward et al., 2006 did not correlate with motor outcome at two years in this cohort. In detail, with regards to Bayley III motor composite score at 20 months, 9 children had scores of less than 85 at 20 months (cohort mean of 96.1 range 61–118) of whom 4 had a GMFCS classification of 2. Only one of these 4 infants had been classified using MRI at the time of birth as having ‘moderate abnormality’ (in this case a score of 10) on MRI at birth using the classification by Woodward et al., 2006. All other infants had a GMFCS classification of 1. In post hoc analysis, there was no correlation between the classification of no/mild/moderate abnormality on MRI at birth and motor outcome: this was true for those classified as having moderate abnormality at term compared with those with no abnormality (*p* = .31) as well as for moderate abnormality compared with mild abnormality (*p* = .49) and mild compared with no abnormality (*p* = .52).

The Bayley cognitive and motor scores were correlated with connectivity at the time of normal birth (Fig. 3). The motor score correlated with the premotor thalamocortical connectivity (Fig. 3, royal blue) at a significance of *p* = < .05 and an effect size (r^2^) of .16. The area showing difference with motor outcome is a large bilateral area of the frontal and medial thalamus in the area of the ventral anterior and ventral lateral nucleus of the thalamus on the left side, extending into the medial and anterior nuclei. On the right side, the area of correlation with outcome excludes the medial-fronto-lateral portion of the thalamus in the area of the ventral anterior nucleus. A unilateral area of left medial thalamus stretching the whole anterior-posterior length of the thalamus is significant at a level of *p* < .01.

Cognitive outcome correlated with the primary sensory-motor thalamocortical connectivity in the right anterior thalamus only (Fig. 3 burgundy colour, Aii). The area is discrete from, but adjacent to, the motor correlations statistic (blue). The contralateral thalamus does not reach significance at *p* < .05, but there is an area of the left thalamus which lies posterior to this position, which approaches significance (*p* < .06). The effect size (r^2^) is .08 which is smaller than that seen for motor outcome. These results are confined to the anterior portion of the thalamus, in the area of the ventral nuclear groups, medial nuclei and anterior nuclei.

## 4 Discussion

In the absence of overt injury, prognostic markers of later child outcome in the preterm born brain are lacking. These results demonstrate the vulnerability of functional thalamocortical connections to the effects of preterm birth and therefore the possibility of using this mechanism to predict outcomes for children who are currently known to be at risk from adverse neurodevelopmental outcome due to prematurity.

### 4.1 Development of thalamocortical connectivity

Thalamocortical connectivity develops largely during the third trimester of pregnancy, the period when children born preterm no longer experience the protective and nurturing intrauterine environment but instead are prematurely exposed to extrauterine life. Cortical projection neurons are generated in the ventricular zone (VZ) and subsequently in the sub ventricular zone (SVZ) (Bystron, Blakemore, & Rakic, 2008) and follow a stereotyped radial pattern of migration leading to the inside out patterning of the neocortex (Angevine & Sidman, 1961; Sauer, 1935). The process of correct topographic patterning requires participation of the cortical sub-plate, a transient structure which hosts thalamocortical projections before they penetrate the cortical plate (Allendoerfer & Shatz, 1994; Bystron et al., 2008; Kostović, Judaš, & Judas, 2010). In human, the subplate reaches maximum thickness between 17 and 24 weeks post-menstrual age, depending on the cortical area investigated, and remains approximately at this thickness until 37 weeks due to the in-growth of fibres from many different systems including basal forebrain fibres, thalamic afferents and axons originating in ipsilateral cortex (Kostovic & Rakic, 1990). The vulnerability of subplate neurons is thought to occur in the form of dysmaturation rather than cell death, with reduced basal dendritic arbor complexity associated with the level of hypoxaemia and metabolic stress in a model of preterm sheep (McClendon et al., 2017). The process of establishing thalamocortical connections appears necessary for subsequent normal morphogenesis to take place (Constantinople & Bruno, 2013; P. O.; Kanold, Kara, Reid, & Shatz, 2003; Li et al., 2013) and is disrupted by preterm birth (Malik et al., 2013).

### 4.2 Neonatal thalamocortical connectivity using magnetic resonance imaging at birth

The disruption to thalamocortical connections in those born preterm has been shown previously using measures from structural magnetic resonance imaging, with reduced thalamic volumes more marked both with increasing prematurity and with diffuse white matter injury (Ball et al., 2011; Boardman et al., 2006; Loh et al., 2017) associated with reduced volume in frontal and temporal lobes (Ball et al., 2011). Connectivity between thalamus and multiple cortical regions was reduced (Ball et al., 2013; Duerden et al., 2019). Altered functional connectivity has also been found between bilateral areas of the thalamus connecting with frontal-parietal-insula cortex and anterior cingulate cortex (Toulmin et al., 2015).

### 4.3 How might preterm birth affect thalamocortical connectivity?

The mechanism of change to thalamocortical circuits is not clear, but reduced arborisation of subplate neurons and the altered environment experienced by the preterm infant may both play a role. Prior to the establishment of sensory information processing, that is, sensory processing based on external stimuli, spontaneous thalamic calcium waves from sensory thalamic nuclei appear to regulate the size of their cortical area in mouse (Moreno-Juan et al., 2017). The onset of central responses to external sensory input has been widely debated in the context of consciousness: even prior to the establishment of definitive circuits, neurons in the subplate are formed as early as the second trimester (Kostovic & Judaš, 2006; Vanhatalo & Kaila, 2006) with neurons in the subplate of ferrets responding to auditory stimuli, as recorded using electrophysiological methods (Wess, Isaiah, Watkins, & Kanold, 2017). The exact timings in response to different sensory modalities are not known, but auropalpebral (‘blink-startle’) reflexes to sound can be seen on ultrasound scans between 24 and 25 weeks gestational age (Birnholz & Benacerraf, 1983). Circuits involving primary cortex, such as primary motor, visual, auditory and sensory cortex, are known to be refined via sensory experience in the time period after the thalamic axons grow into cortical layer IV (Allendoerfer & Shatz, 1994; Friauf & Shatz, 1991; Patrick O.; Kanold & Shatz, 2006), that is during the third trimester, giving rise to the suggestion that abnormal auditory, sensory and painful experiences of the preterm infant might have an effect on the balance of these circuits (Duerden et al., 2018; Slater et al., 2012). With global connections and involvement in multiple cognitive functions (Hwang, Bertolero, Liu, & D'Esposito, 2017), the thalamus has been shown to be a critical hub for information processing and integration, with an intact system of thalamocortical connectivity in adults proving essential for maintaining cognitive performance (Hughes et al., 2012; Hwang et al., 2017; Ystad et al., 2011).

### 4.4 How do changes in functional connectivity noted at birth relate to performance at two years old

We found that thalamic connectivity to Primary Sensory-Motor and Motor Association components was correlated with neurocognitive outcome. The Primary Sensory-Motor component does not include the whole primary sensory-motor cortex, but only the medial portion, containing areas concerned with sensation and motor control of limbs and trunk but excluding those concerning swallowing, tongue and face. Primary sensory-motor cortex concerning swallowing, tongue and face was not included in the analysis as this network split between two separate components in this group at the time of normal birth. The motor association component encompasses both the premotor cortex and the supplementary motor area. The location of the difference in connectivity according to outcome score is in the medial and anterior portion of the thalamus and although specific nuclei cannot be defined, this is the location of inputs from the globus pallidus internal segment and substia nigra of the basal ganglia (Mai & Paxinos, 2012) which sends direct efferents to the premotor cortex including the supplementary motor cortex (Morel, Liu, Wannier, Jeanmonod, & Rouiller, 2005; Strick, 1976) and to the primary motor cortex (Kultas-Ilinsky, Sivan-Loukianova, & Ilinsky, 2003). This motor area of the thalamus is part of the cortico-striatal-thalamo-cortical circuits described by Alexander et al. (Alexander, DeLong, & Strick, 1986) which help to prompt, enact and guide different aspects of voluntary movements (Middleton & Strick, 2000, 2002) and are essential for motor learning (Tanaka et al., 2018).

### 4.5 How successful are functional connectivity estimates with regards to outcome at two years

These difference in connectivity accounted for 16% of the variance in motor score and 8% in cognitive score. The most comparable cohort with data available with which to make a comparison, finds mean thalamocortical connectivity across the whole cortex explains 11% of the variance in cognitive scores at two years, with the addition of socio-economic status increasing this to 30% (Ball et al., 2015). With regards to the Bayley motor scale itself, when assessing preterm children without cerebral palsy, as in this study, it has a predictive value for motor functioning at early school age of *r* = .34, equating to an explained variance of 12% (Luttikhuizen dos Santos, de Kieviet, Königs, van Elburg, & Oosterlaan, 2013). It is often commented that preterm infants have performed within the normal ranges stated in the Bayley III tests but that their scores are significantly lower than their term counterparts (Bapat, Narayana, Zhou, & Parikh, 2014; Kendall et al., 2014; Skiöld et al., 2012). This may indicate that the difficulties in multiple domains experienced by children born preterm is not well-captured by the scoring system of the Bayley III which, performed at two years, underestimates motor deficits by the age of four (Spittle et al., 2013).

### 4.6 Why are motor areas affected in preterm infants?

Is it possible that success in many neurodevelopmental tests at two years old is mediated by motor skills, but impairments to motor circuits are also involved in disorders which have been more traditionally thought to be behavioural or psychiatric and the results presented here may be early markers of non-motor impairments. In addition to an increased risk of autism and anxiety disorders (D'Onofrio et al., 2013; Treyvaud et al., 2013), preterm infants are at increased risk of behavioural difficulties such as attention deficit hyperactivity disorder (Delobel-Ayoub et al., 2006; Jaekel, Wolke, & Bartmann, 2013; Månsson, Stjernqvist, & Bäckström, 2014; Marret et al., 2013; Spittle et al., 2009; Treyvaud et al., 2013) where disordered development of motor skills are seen (Mostofsky et al., 2006). Motor delays are often the first area of concern (mean age of 14.7 months) reported by parents of children with Autism Spectrum Disorders (Chawarska et al., 2007) and lower gross motor function (Estes et al., 2015; Lloyd, MacDonald, & Lord, 2013; Marrus et al., 2018) are early risk markers. Others identify difficulty with motor planning and impaired fine motor control as the most prevalent and earliest identifiable motor deficits in children with autism (Fournier, Hass, Naik, Lodha, & Cauraugh, 2010; Green et al., 2009; Teitelbaum, Teitelbaum, Nye, Fryman, & Maurer, 1998). Indeed, sensorimotor difficulties, defined as ‘an impairment in the pathway involving motor activity triggered by sensory stimuli and repetitive motor movements’ are included in the diagnostic criteria for autism (American Psychiatric Association., & American Psychiatric Association. DSM-5 Task Force, 2013). It has also been hypothesised that difficulties with motor coordination and sensory difficulties may underlie the social and communication deficits, for example impairments in skilled motor gestures (Mostofsky et al., 2009).

### 4.7 Limitations

This work has some limitations. There are challenges in acquiring excellent quality functional MRI data in infants, and chloral hydrate sedation was used as it has been in many other studies (Brouwer et al., 2017; Kidokoro et al., 2014; Kidokoro, Neil, & Inder, 2013). This may be a limitation however it is not clear that sedation affects resting state fMRI data: oculomotor, somatomotor, visual and default mode networks have all been shown to be coherent even under anaesthetic levels which induce a profound loss of consciousness (Vincent et al., 2007). Indeed, changes of vigilance and arousal in adult subjects during the course of a scan may have a greater effect (Deco, Hagmann, Hudetz, & Tononi, 2014; Massimini et al., 2005; Saper, Scammell, & Lu, 2005). Acknowledging the sensitivity of functional data to motion, (for a review of the issues see Murphy et al., 2013; Van Dijk et al., 2012), and with the aim of investigating small subcortical structures in infants, only datasets with very low motion were eligible for inclusion. A limit of a relative mean displacement of .08 mm was chosen, but additional data sets which met this criterion but contained absolute motion of >2 mm were excluded as there can be a prolonged effect from a single episode of motion (Power et al., 2014). The inclusion of only the bilateral cortical regions of interest, whilst allowing an unbiased method for selecting cortical regions of interest for use in analysis, does not allow for analysis of all cortical regions. This methodology was adopted to avoid making a priori assumptions about neonatal function based on knowledge of the functions of the mature brain. Also, as poor motor scores at two years old do not capture all infants who will go on to have motor difficulties, it will be important to test functional connectivity with motor outcome scores in older, un-sedated children as data becomes available.

## 5 Conclusion

This study shows altered connectivity between cortical areas and thalamus in infants born very preterm and relates this to their abilities at two years old as assessed by a standardised neurodevelopmental assessment. We find differences in anatomically plausible corticothalamic pairings associated with increased difficulties with motor and cognitive outcomes at two years old. Future work should investigate the outcome of these children at a later age and investigate whether early interventions based on these metrics gathered at the time of normal birth could support the development of at-risk children.

## CRediT author statement

Hilary Toulmin, Conceptualization; Formal analysis; Funding acquisition; Methodology; Roles/Writing–original draft;

Jonathan O'Muircheataigh, Conceptualization; Formal analysis; Methodology; Supervision; Roles/Writing–original draft;

Serena J. Counsell, Conceptualization; Investigation; Supervision; Writing–review & editing.

Shona Falconer, Investigation; Writing–review & editing.

Andrew Chew, Investigation; Writing–review & editing.

Christian F. Beckmann, Conceptualization; Formal analysis; Methodology; Software; Supervision; Roles/Writing–original draft.

A. David Edwards. Conceptualization; Funding acquisition; Investigation; Methodology; Project administration; Supervision; Roles/Writing–original draft.

## Data availability

The conditions of our ethics approval do not permit public archiving of anonymised study data. Readers seeking access to the data should contact the lead author Professor Edwards at the Centre for the Developing Brain, King's College, London. Access will be granted to individuals in accordance with our ethical approvals. Specifically, an application to the National Research Ethics Committee is likely to be required to allow access.

## Analysis code used

From FSL as referenced in the methods section.

## Preregistration

No part of the study procedures was pre-registered prior to the research being conducted. The collection of data for ePrime, a parallel-group randomised trial, was pre-registered as a trial on https//clinicaltrials.gov/ct/show/NCT01049594.

## Funding information

This research uses data from research commissioned by the 10.13039/501100000272National Institute for Health Research (NIHR) Programme Grants for Applied Research Programme (RP-PG-0707-10,154) (ePrime). The views and opinions expressed by authors in this publication are those of the authors and do not necessarily reflect those of the National Health Service (NHS), the NIHR, Medical Research Council, Central Commissioning Facility, NIHR Evaluation, Trials and Studies Coordinating Centre, the Programme Grants for Applied Research programme or the Department of Health. H.T. is funded by Wellcome Trust Research Training Fellowship 096039. J.O.M. is supported by a Sir Henry Dale Fellowship jointly funded by the Wellcome Trust and the Royal Society (206675/Z/17/Z). This research was supported by: Wellcome EPSRC Centre for Medical Engineering at King's College London (WT 203148/Z/16/Z); the National Institute for Health Research Biomedical Research Centre based at Guy's and St Thomas' NHS Foundation Trust and King's College London; and the MRC Centre for Neurodevelopmental Disorders (MR/P502108/1).

## Declaration of competing interest

The authors have no competing interests to declare.
